# The Association Between Body Fat Percentage and Severe Headache or Migraine: A National Cross-Sectional Survey

**DOI:** 10.7759/cureus.72446

**Published:** 2024-10-26

**Authors:** Rongjiang Xu, Liang Dong, Xiaonuo Xu, Xiaoping Fan, Jiying Zhou

**Affiliations:** 1 Department of Neurology, The First Affiliated Hospital of Chongqing Medical University, Chongqing, CHN; 2 Phase I Clinical Research Center, The First Affiliated Hospital of Chongqing Medical University, Chongqing, CHN

**Keywords:** body fat percentage, cross-sectional study, national health and nutrition examination survey, obesity, severe headache or migraine

## Abstract

Background and objective

Migraine is characterized by recurrent headaches, frequently associated with nausea, photophobia, and phonophobia; it is highly prevalent and linked to a heavy socioeconomic burden. While the prevalence of obesity in the general population has increased in recent years, no prior studies exist regarding the relationship between body fat percentage (BF%) and the incidence of severe headaches or migraine. In light of this, we conducted this study to address this gap in data.

Methods

We utilized data from the National Health and Nutrition Examination Survey (NHANES) involving 5,060 individuals (1999-2004). After adjusting for gender, poverty-to-income ratio (PIR), educational attainment, smoking status, moderate physical activity, and hypertension, we employed restricted cubic spline (RCS) curves and logistic regression to examine the relationship between BF% and the occurrence of severe headaches or migraine.

Results

The study included 5,060 participants: 1,289 (25.5%) with severe headache or migraine and 3,771 (74.5%) without. Compared to patients without severe headaches or migraine, those with it were more likely to be female and have lower educational attainment, household income, and physical activity, as well as smoking, alcohol consumption, diabetes, and hypertension. In females in particular and overall, the models showed a significant association between BF% and severe headache or migraine, whereas there was no association between BF% and severe headache or migraine in the male population. Multivariate logistic regression analyses using BF% quartiles yielded similar results.

Conclusions

We observed a substantial positive correlation between BF% and severe headaches or migraine after controlling for pertinent variables. This correlation was particularly strong in females. These findings underscore the intricate nature of the relationship between obesity and migraine, highlighting the necessity for more investigation to clarify the role that BF% plays in the escalation of migraine.

## Introduction

Migraine is a debilitating, intricate neurological condition characterized by an attack duration of 4-72 hours, unilateral localization, pulsing nature, moderate to severe intensity, and accompanying symptoms such as photophobia, nausea, and vomiting [1]. Migraine is the largest cause of disability among women aged 15-49 years, and it is the second leading cause of disability across all age groups globally [2,3].

The incidence of overweight and obesity has significantly increased in recent decades and is currently a primary risk factor for morbidity and mortality worldwide [4]. A previous review showed that overweight or obese individuals are more likely to suffer from migraines than normal-weight individuals [5]. Another study demonstrated a positive relationship between obesity and the prevalence of migraine with aura, revealing that persons with obesity experienced a greater frequency of daily headaches than those of normal weight [6]. Young female patients with high body fat percentage (BF%) are at increased risk of developing chronic migraine, and weight control measures can be targeted to prevent migraine worsening [7]. Hence, it is critical to investigate the correlation between obesity and migraine in the general population.

Although few studies have shown an association between BF% and migraine, it is important to recognize that BF% may individually influence the onset of migraine. This study hypothesized that as BF% increases, the risk of developing migraine also increases. Therefore, the purpose of this study was to investigate the relationship between BF% and the risk of migraine occurrence. This study analyzed data from the National Health and Demographic Survey database (NHANES) to address the lack of knowledge regarding migraine and obesity, and to provide some guidance on weight control in obese migraineurs.

## Materials and methods

Study population

Our data is derived from NHANES, an ongoing investigation of the American population sponsored by the National Center for Health Statistics (NCHS). It contains a wealth of details on the diet and overall health of Americans. NHANES utilizes a well-represented sample since the study is conducted every two years and employs a stratified, multiple-step probability sampling technique. The NCHS Ethics Committee approved the research methodologies and informed consent forms before gathering data kicked off. All information can be obtained at https://www.cdc.gov/nchs/nhanes/.

This research was based on the 1999-2004 NHANES survey cycle, the only one to include a question on severe headaches or migraine. A total of 31,126 qualified individuals were originally selected, and after eliminating 15,798 participants with missing data on severe headache or migraine and 10,268 people with incomplete data on BF%, the remainder of the participants were all adults over the age of 20 years. Ultimately, 5,060 participants were included in this study.

Assessment of body fat percentage

The BF% was calculated using bioelectrical impedance analysis (BIA) on the NHANES datasets. BIA is a technique that evaluates body fluid volumes, total body water, and fat-free mass by measuring the electrical impedance of bodily tissues. As part of the Body Composition component of the Mobile Examination Center (MEC), the NHANES bio-impedance spectroscopy (BIS) multi-frequency measurements were gathered in the BIA examination together with dual-energy X-ray absorptiometry (DXA) images.

Outcome variable

A part of the NHANES survey called "Miscellaneous Pain (MPQ)" was used to find out about migraine: (MPQ090): "Have you had severe headaches or migraines in the last three months?". These people were thought to have migraines when they said "yes." Most of the people who had bad headaches probably had migraines, but NHANES could not tell them what kind of headache or migraine they had. This study from the American Migraine Frequency and Prevention (AMPP) used data from the 2004 U.S. Migraine Prevalence and Prevention Study to show how often migraines, probable migraines (PM), and other serious headaches happen; 11.8% of the people who answered had a migraine, 4.6% had a stress headache, and 17.4% had a "severe headache." The International Classification of Headache Disorders-2 was used to come up with these numbers. Only 1% of the cases had "other severe headaches" written in [8].

Covariates

A variety of variables was carefully evaluated. The demographic factors included age, race, gender, educational levels, marital status, and poverty-to-income ratio (PIR). Race was categorized into five groups: non-Hispanic white, non-Hispanic black, Mexican American, other Hispanic, and others. Educational levels were classified into three categories: below high school, high school, and more than high school. Smoking status was categorized as never, former, or current, utilizing SMQ020, which assesses whether an individual has smoked at least 100 cigarettes in their lifetime, and SMQ040, which inquires if the individual currently smokes cigarettes. Marital status was classified as either cohabiting with a partner or living alone.

Household income was categorized according to PIR into three levels: low (PIR ≤1.3), moderate (PIR >1.3 to 1.85), and high (PIR >1.85). Alcohol consumption was categorized as present or absent based on the intake of a minimum of 12 alcoholic beverages within a year. Activity level was defined by the presence or absence of a moderate level of activity. Hypertension diagnosis was based on three blood pressure readings exceeding 140/90 mmHg on separate days, in conjunction with a previous diagnosis or the administration of antihypertensive medication. Diabetes was defined by a prior diagnosis, the administration of glucose-lowering medications or insulin, fasting blood glucose levels of ≥126 mg/dL, or HbA1c levels exceeding 6.5%.

Statistical analysis

Analytic Guidelines for NHANES Complex sampling weight calculations ensured appropriate weighting of sample data. Participant characteristics for continuous variables that followed a normal distribution were summarized using mean ± standard deviation (SD), while percentages were employed for categorical variables. Differences between groups were evaluated using analysis of variance (ANOVA) for continuous variables and chi-square tests for categorical variables. Initial one-way logistic analyses were conducted to eliminate variables that lacked both statistical and clinical significance. In multivariate logistic analyses, only screened variables were included. Following the adjustment for potential confounders, multivariate logistic regression models produced odds ratios (ORs) and 95% confidence intervals (CIs) regarding the relationship between BF% and the risk of severe headache or migraine. Due to the minimal impact of each 1% increase in body fat on migraine, we categorized BF% into quartiles and treated them as continuous variables, designating the second quartile as the reference group for multivariate logistic regression analysis.

In addition, we performed multiple restricted cubic spline (RCS) curve analyses to explore the nonlinear dose-response relationship between BF% and migraine risk in the whole population. Because of the gender imbalance in migraine, we made a stratified analysis by gender. Statistical analyses were conducted using R version 4.4.0, with statistical significance set at a two-sided p-value of 0.05.

## Results

The demographic characteristics of the entire population included in this study are detailed in Table 1. Of the 5,060 participants enrolled in NHANES from 1999 to 2004, 51.5% (n = 2,605) were male and 48.5% (n = 2,455) were female. A total of 1,289 individuals were diagnosed with severe headaches or migraine, representing 25.5% of the entire sample. Compared to those without severe headaches or migraine, those with severe headaches or migraine were more likely to be female, less educated, have lower family incomes, and less moderately active; and have higher rates of smoking and alcohol consumption, diabetes, and high blood pressure. In addition, BF% was higher in people with severe headaches or migraine compared to those without [34.89 (10.34) in the migraine group and 30.49 (10.61) in the non-migraine group, p<0.001)].

**Table 1:** Characteristics of the study participants Q1: 0-23.6; Q2: 23.6-31.8; Q3: 31.8-39.4; Q4: 39.4-64.2 BFP: body fat percentage; PIR: poverty-to-income ratio; SD: standard deviation

Characters	Total	Severe headache or migraine		P-value
		No	Yes	-
-	5,060	3,771	1,289	-
Gender, n (%)	-	-	-	-
Female	2,455 (48.5)	1,600 (42.4)	855 (66.3)	<0.001
Male	2,605 (51.5)	2,171 (57.6)	434 (33.7)	-
Age, years, mean (SD)	34.57 (8.76)	34.46 (8.81)	34.89 (8.60)	0.129
PIR, n (%)	-	-	-	-
High income	2,740 (58.2)	2,152 (61.3)	588 (49.2)	<0.001
Low income	1,349 (28.7)	925 (26.4)	424 (35.5)	-
Medium income	615 (13.1)	432 (12.3)	183 (15.3)	-
Race, n (%)	-	-	-	-
Mexican American	1,302 (25.7)	975 (25.9)	327 (25.4)	0.409
Non-Hispanic black	1,082 (21.4)	791 (21.0)	291 (22.6)	-
Non-Hispanic white	2,253 (44.5)	1,694 (44.9)	559 (43.4)	-
Other Hispanic	260 (5.1)	185 (4.9)	75 (5.8)	-
Others	163 (3.2)	126 (3.3)	37 (2.9)	-
Marital status, n (%)	-	-	-	-
Living alone	1,993 (40.5)	1,489 (40.5)	504 (40.7)	0.898
Married or living with a partner	2,926 (59.5)	2,192 (59.5)	734 (59.3)	-
Alcohol consumption, n (%)	-	-	-	-
No	1,266 (26.3)	874 (24.4)	392 (32.0)	<0.001
Yes	3,542 (73.7)	2,710 (75.6)	832 (68.0)	-
Smoking status, n (%)	-	-	-	-
Current	1,473 (29.1)	1,049 (27.8)	424 (32.9)	0.002
Former	780 (15.4)	594 (15.8)	186 (14.4)	-
Never	2,803 (55.4)	2,125 (56.4)	678 (52.6)	-
Moderate activity, n (%)	-	-	-	-
No	2,611 (51.6)	1,908 (50.6)	703 (54.5)	0.016
Yes	2,449 (48.4)	1,863 (49.4)	586 (45.5)	-
Hypertension, n (%)	-	-	-	-
No	4,259 (85.6)	3,227 (87.2)	1,032 (81.0)	<0.001
Yes	716 (14.4)	474 (12.8)	242 (19.0)	-
Diabetes, n (%)	-	-	-	-
No	4,826 (95.4)	3,615 (95.9)	1,211 (93.9)	0.006
Yes	234 (4.6)	156 (4.1)	78 (6.1)	-
BFP, mean (SD)	31.61 (10.71)	30.49 (10.61)	34.89 (10.34)	<0.001
BFP quantile, n (%)	-	-	-	-
Q1	1,273 (25.2)	1,067 (28.3)	206 (16.0)	<0.001
Q2	1,249 (24.7)	998 (26.5)	251 (19.5)	-
Q3	1,210 (23.9)	874 (23.2)	336 (26.1)	-
Q4	1,328 (26.2)	832 (22.1)	496 (38.5)	-

**Figure 1:**
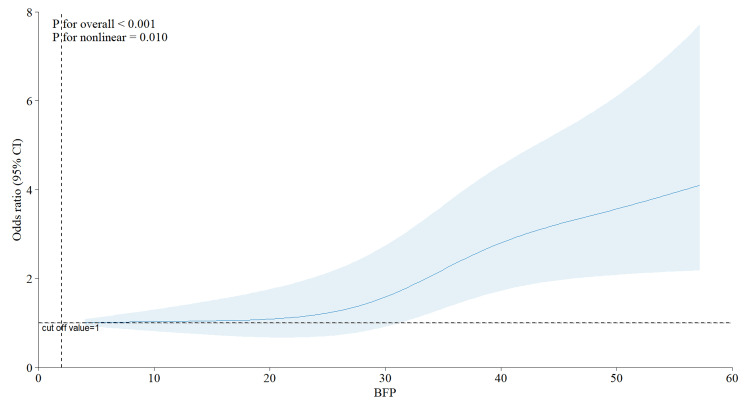
The relationship between body fat percentage and severe headache or migraine Adjusted for gender, PIR, education levels, smoke status, moderate activity, and hypertension BFP: body fat percentage; PIR: poverty-to-income ratio

Figure 1 illustrates a positive nonlinear connection between BF% and migraine (non-linearity p = 0.01). The risk of migraine exhibited a slow rise until the BF% approached 30, after which it escalated sharply, with no further decline observed. Therefore, BF% was categorized into quartiles for further study, with Q2 (23.6-31.8%) designated as the reference group. As previously mentioned in the Materials & Methods section, we included only those variables that exhibited statistical significance in univariate logistic regression for the multivariate logistic regression analyses, ultimately incorporating gender, PIR, educational attainment, smoking status, moderate physical activity, and hypertension (refer to the table in the appendices for details). 

**Figure 2:**
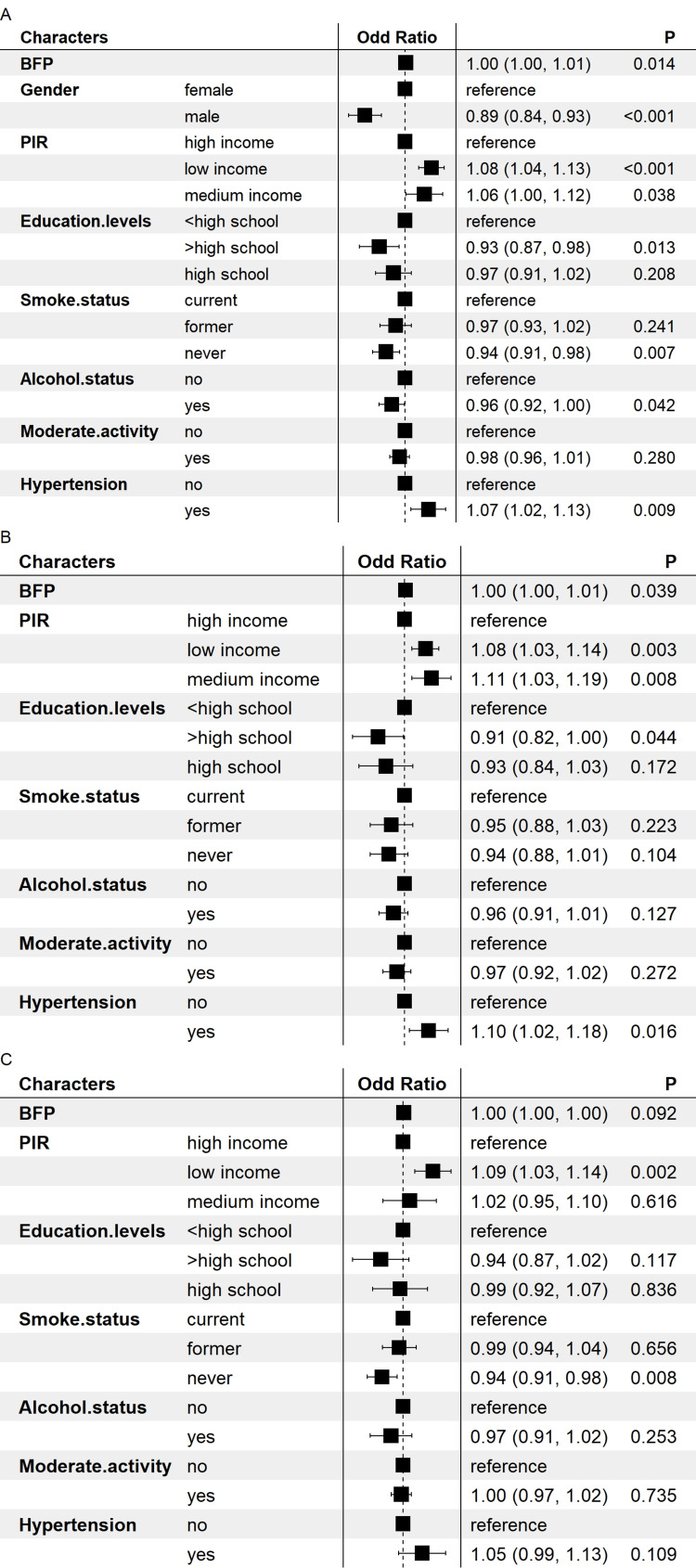
Forest plot of the association between body fat percentage and severe headache or migraine Adjusted for gender, PIR, education levels, smoke status, moderate activity, and hypertension. A: the whole group; B: the female group; C: the male group BFP: body fat percentage; PIR: poverty-to-income ratio

As illustrated in Figure 2, we conducted a multivariate logistic regression analysis using BF% as a continuous variable. Before the gender subgroup analysis, the model indicated a positive correlation between BF% and severe headache or migraine (OR: 1.00-1.01, p = 0.014). Additionally, being male, having a lower PIR, higher educational attainment, reduced smoking, alcohol consumption in the past year, and the absence of hypertension were negatively correlated with severe headaches or migraine. Moreover, we conducted a gender-stratified analysis and discovered that BF% exhibited a positive correlation with severe headache or migraine in females (OR: 1.00-1.01, p = 0.039). Furthermore, reduced PIR, higher educational attainment, and the absence of hypertension were inversely correlated with severe headache or migraine; in the male subgroup, the model indicated no correlation between BF% and severe headache or migraine.

**Figure 3:**
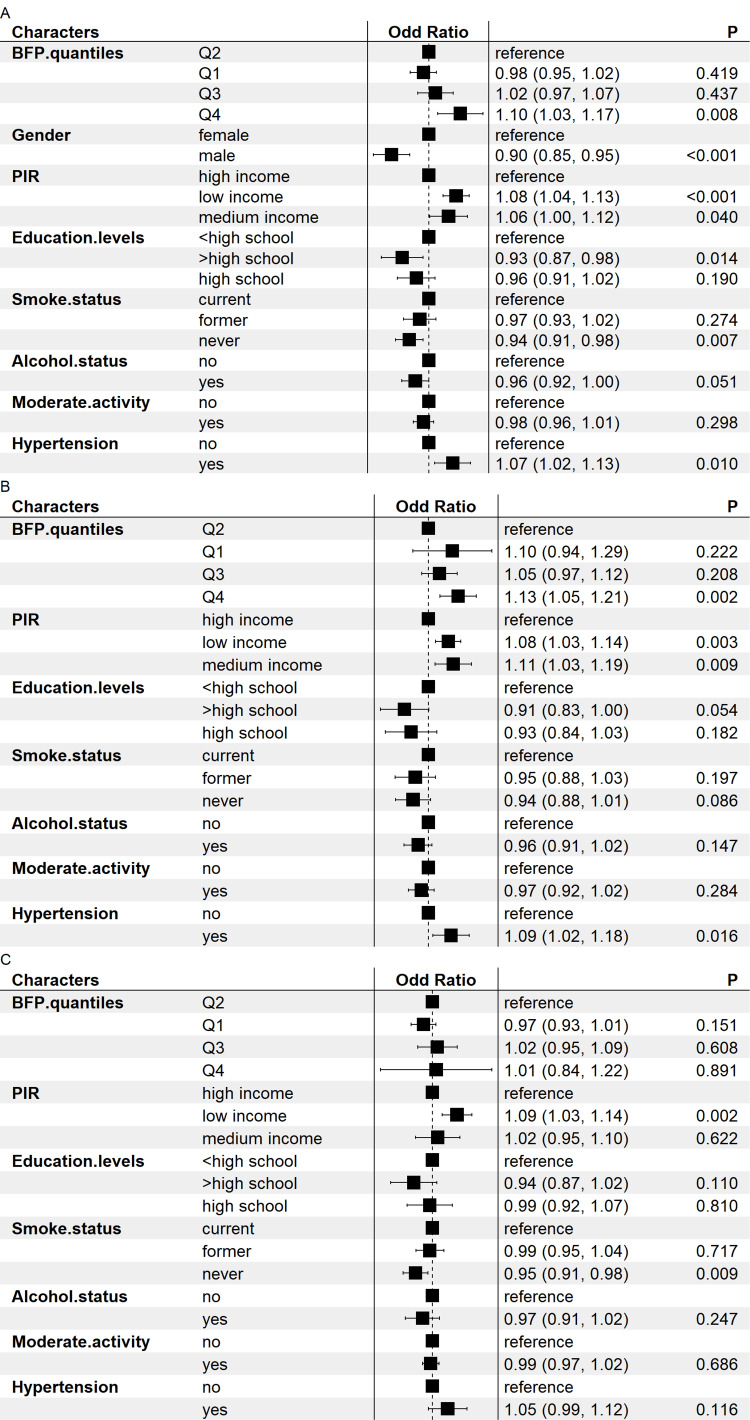
Forest plot of association between body fat percentage quantiles and severe headache or migraine Q1: 0-23.6; Q2: 23.6-31.8; Q3: 31.8-39.4; Q4: 39.4-64.2; A: the whole group; B: the female group; C: the male group BFP: body fat percentage; PIR: poverty-to-income ratio

With Q2 as the reference group for multivariate statistical regression analysis, we further split BF% into quartiles, as seen in Figure 3. Before subgroup analyses by gender, the model indicated a significant correlation between BF% and the incidence of severe headache or migraine (OR Q4 vs. 2 = 1.10, 95% CI: 1.03-1.17). Moreover, male gender, lower PIR, better educational attainment, reduced smoking, and absence of hypertension were inversely correlated with severe headaches or migraine. Furthermore, we conducted a sex-stratified analysis and discovered that BF% in the female cohort was strongly associated with severe headache or migraine (OR Q4 vs. 2 = 1.13, 95% CI: 1.05-1.21). Moreover, reduced PIR values, higher educational attainment, and the lack of hypertension exhibited an inverse relationship with severe headaches or migraine; among the male subgroup, the model indicated no link between BF% and severe headaches or migraine.

## Discussion

This study represents the first large-scale cross-sectional analysis examining the relationship between BF% and headache prevalence utilizing the NHANES database. The results indicate a notable positive correlation between BF% and headache occurrence, particularly among females. Furthermore, the RCS curve indicates that the risk of migraine escalates with increasing BF%, implying a possible association between obesity and migraine occurrence.

BMI is one of the most commonly used methods for estimating obesity, and the association with migraine has been examined in several previous studies. Although BMI is easy to measure and useful in clinical research, it is not an excellent predictor of obesity and obesity-related illnesses, which does not directly assess body composition and does not predict obesity in those without extra fat mass [9]. Also, BMI does not discriminate between visceral and subcutaneous fat [9]. This is essential since body fats affect illness risk differently. Then BIA is a highly regarded parameter due to its simplicity and absence of radiation in determining BF%.

The relationship between obesity and migraine has been previously examined; however, research has predominantly concentrated on BMI due to prior complaints, resulting in limited investigation into BF% in relation to migraine. A study involving 168 premenopausal female patients with episodic migraine and chronic migraine found that premenopausal patients with high BF% of episodic migraine may be at an increased risk of developing chronic migraine and that appropriate measures to promote weight control are needed [7]. Estimating obesity using BF% showed a significant association between adiposity and migraine chronicity [7]. A study evaluating the prevalence of migraine and severe headache in individuals with and without generalized and abdominal obesity found that the relationship between migraine and obesity differed based on age, gender, and adipose tissue distribution [10].

Another study assessing BMI, waist circumference, BF%, and migraine-related clinical variables in 166 female migraineurs aged ≥18 years revealed weak correlations between BMI and waist circumference with attack frequency over six months [11]. To investigate the association between obesity and migraine, a neurologist clinically evaluated 684 women between the ages of 40-74 and found no significant association between migraine or migraine characteristics and obesity [12]. The varying results observed across studies may stem from the use of distinct migraine diagnostic criteria and differences in the clinical and demographic characteristics of the populations studied. This underscores the necessity for a large-scale investigation involving diverse populations.

Previous studies have shown that obesity is a risk factor for migraine, advising patients that losing body fat may yield benefits. The treatment of migraine in overweight or obese patients is complicated by the fact that some medications for preventing headache attacks, for example, cause weight gain [13]. Therefore, indications for migraine therapeutic agents that induce weight loss would improve patient compliance with prophylactic treatment. Topiramate is one of the approved migraine prophylactic medications, and it reduces the frequency of migraine attacks while reducing body weight, making it an ideal prophylactic medication for patients with chronic migraine and obesity [14]. A study involving 26 female chronic migraine patients between the ages of 18 and 45 years who received three months of prophylactic treatment with topiramate (50 mg/day) showed that in addition to decreasing body fat, topiramate led to an increase in fat-free mass (FFM) in obese patients [15]. Other studies have also reached similar conclusions, with a reduction in body fat in migraine patients treated with topiramate.

Recently, adolescent obese migraineurs who underwent a non-surgical weight loss intervention program demonstrated significant improvements in obesity, migraine frequency, intensity, and disability on the MIDAS Disability Assessment Scale (MIDAS), as well as in the use of post-treatment headache medications [16]. In addition, the symptoms of migraine patients improve after surgical weight loss. A study comparing 54 morbidly obese female patients with migraine who were enrolled in a bariatric clinic for either BS [vertical sleeve gastrectomy (VSG) (n = 25)] or behavioral therapy (BT) found that although non-surgical modalities (BT) including diet and exercise had an effect on migraine severity and the number of migraine days, their effect was reduced [17] and it was not comparable with BS [17]. Most of these findings further support the conclusions of this study.

The relationship between migraine and obesity remains ambiguous; however, numerous prior studies have provided valuable insights. According to the findings from preclinical and clinical studies, several potential mechanisms have been suggested to clarify the relationship between migraine and obesity. These include the heightened release of proinflammatory substances, neuroinflammatory neuropeptides, and the metabolism of adipose tissue. Proinflammatory substances significantly contribute to several pathological processes, including inflammation and pain. Elevated inflammatory factors in obese persons, compared to those of normal weight, may exacerbate migraine episodes [18].

Migraine sufferers (with and without aura) and obese patients have elevated plasma or serum concentrations of many inflammatory markers, including IL-1β, IL-6, IL-8, TNF-α, and C-reactive protein levels [19]. Moreover, calcitonin gene-related peptide (CGRP) appears to influence both migraine and obesity. Serum CGRP levels are increased in persons with migraines and those who are fat [20,21]. CGRP is implicated in the onset of throbbing headaches and migraine-associated symptoms [22], while in obesity, dietary fat may stimulate CGRP production [21]. Adipokines related to lipid metabolism have also been linked to migraine. Lipocalin and leptin are adipokines mostly secreted by subcutaneous adipose tissue [23]. Both lipocalin and leptin are elevated between migraine episodes but may be diminished during attacks [24].

This study has several limitations. It focused on the adult demographic in the United States, specifically individuals aged 20 years older. To find out if our results hold for other age groups and demographics in other countries and regions, more research is required. Furthermore, the data regarding severe headaches or migraine were not subjected to clinical diagnosis by specialists in the field of headaches. Instead, they were collected using self-report questionnaires. The identification of migraine was based solely on a single question asking whether the individual had experienced a severe headache or migraine within the last three months. Also, there was a lack of information about the severity of the intense headache or migraine, any other symptoms experienced, or the specific subtype of migraine affecting the subjects. Hence, the task of analyzing several subtypes of migraine and their respective associated symptoms individually poses a challenge. Moreover, the use of a dependable dietary recall approach in this study to evaluate overall food consumption may have led to memory bias or reporting bias. Since this study was ultimately cross-sectional, it was unable to determine causal links. We recommend prospective cohort studies to obtain more accurate findings.

## Conclusions

This study demonstrated a significant positive correlation between BF% and total migraine incidence. This finding is key for comprehending the association between obesity and migraine, indicating that migraine prevention may be achievable by reducing BF% through exercise, medication, or surgical intervention. Maintaining appropriate levels of BF% is essential. We believe our findings will improve our capacity to implement timely and targeted interventions, thereby enhancing the quality of life for individuals with migraines.

## Appendices

**Table 2:** Weighted univariate logistic regression results Q1: 0-23.6; Q2: 23.6-31.8; Q3: 31.8-39.4; Q4: 39.4-64.2 BFP: body fat percentage; CI: confidence interval; OR: odds ratio; PIR: poverty-to-income ratio; SD: standard deviation

Variables		OR (95% CI)	P-value
				Gender, n (%)	Female	Ref	-
-	Male	0.84 (0.82-0.87）	<0.01
Age, mean (SD)	-	1.00 (1.00-1.00）	0.56
PIR, n (%)	High income	Ref	-
-	Low income	1.13 (1.09-1.18)	<0.01
-	Medium income	1.09 (1.03-1.14)	<0.01
Race, n (%)	Mexican American	Ref	-
-	Non-Hispanic black	1.04 (0.99-1.08)	0.09
-	Non-Hispanic white	1.00 (0.97-1.04)	0.82
-	Other Hispanic	1.05 (0.98-1.13)	0.15
-	Others	0.99 (0.91-1.07)	0.81
Marital status, n (%)	Living alone	Ref	-
-	Married or living with a partner	1.01 (0.99-1.03)	0.4
Alcohol consumption, n (%)	No	Ref	-
-	Yes	0.93 (0.90-0.96)	<0.01
Smoking status, n (%)	Current	Ref	-
-	Former	0.95 (0.90-0.99)	0.02
-	Never	0.94 (0.90-0.98)	<0.01
Moderate activity, n (%)	No	Ref	-
-	Yes	0.96 (0.94-0.99)	0.01
Hypertension, n (%)	No	Ref	
-	Yes	1.08 (1.03-1.33)	<0.01
Diabetes, n (%)	No	Ref	-
-	Yes	1.05 (0.98-1.11)	0.14
BFP, mean (SD)	-	1.008 (1.006-1.009)	<0.01
BFP quantile, n (%)	Q2	Ref	-
-	Q1	1273 (25.2)	0.03
-	Q3	1210 (23.9)	<0.01
-	Q4	1328 (26.2)	<0.01
